# Supplementary figures and images for: The role of interleukin-6 as a prognostic biomarker for predicting acute exacerbation in interstitial lung diseases
Source: PLoS One. 2021 Jul 27;16(7):e0255365. doi: 10.1371/journal.pone.0255365 (PMC8315549; doi:10.1371/journal.pone.0255365)

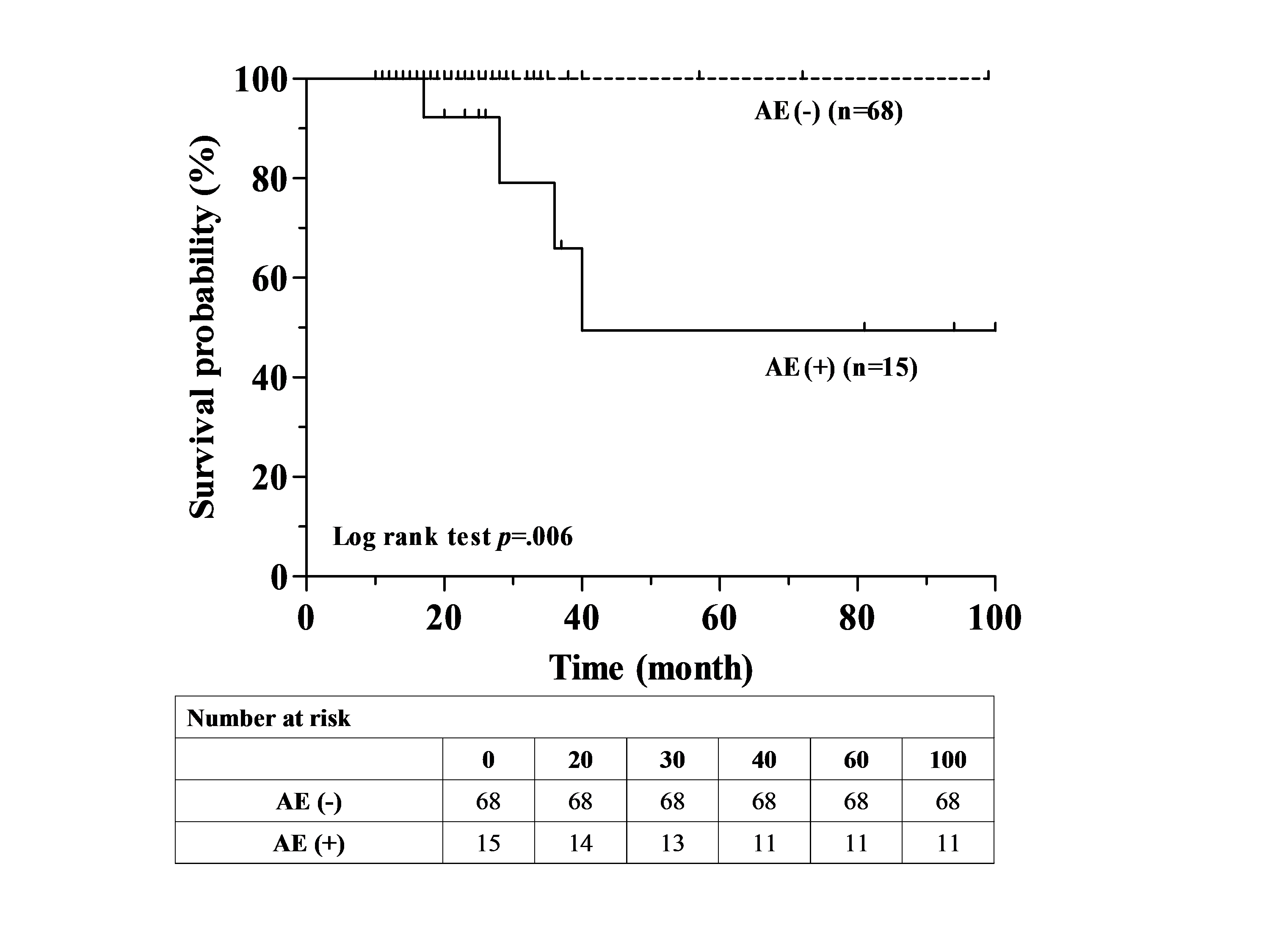

Supplement: S1 Fig — (TIF) [file pone.0255365.s001.tif]
