# Supplementary material for: The role of interleukin-6 as a prognostic biomarker for predicting acute exacerbation in interstitial lung diseases
Source: PLoS One. 2021 Jul 27;16(7):e0255365. doi: 10.1371/journal.pone.0255365 (PMC8315549; doi:10.1371/journal.pone.0255365)
Supplement: Attachment — Submitted filename: Response to Reviewers.docx [file pone.0255365.s002.docx]

May 15, 2021

Minghua Wu

PLOS ONE

Submission ID: PONE-D-21-04098

Manuscript title: The Role of Interleukin-6 as a Prognostic Biomarker for Predicting Acute Exacerbation in Interstitial Lung Diseases

Dear Editor,

We would like to thank you for the opportunity to resubmit a revised copy of our manuscript. We would also like to take this opportunity to express our thanks to the reviewers for the positive feedback and helpful comments for correction or modification.

We have made substantial changes to the manuscript according to the reviewers’ comments. The changes made are marked in red in the revised manuscript. Our point-by-point responses to the reviewers' comments are provided below. We hope the revised manuscript is suitable for publication in *PLOS ONE.*

Sincerely,

Hyun Kuk Kim, M.D., Ph.D.

Department of Pulmonary and Critical Care Medicine, Inje University Haeundae Paik Hospital, Inje University College of Medicine, Haeundae-ro 875, Haeundae-gu, 48108, Busan, Republic of Korea.

Tel: +82-51-797-2210, Fax: +82-51-797-2211, E-mail: khkmd1205@hanmail.net

**Reviewer Comments**

Q1. Table 1 & 2: Add % to the patient characteristic population ratio even explained at the bottom of the table, for example,  IPF, n (%)--- 57 (68.7).

R1. Thank you for pointing this out. Based on this comment, we add % in the Table 1 & 2 as below.

[RESULTS, Study population]

**Table 1. Baseline Clinical Characteristics of the Patients**

| **Characteristics** | **All Patients (n = 83)** |
| --- | --- |
| Age, years | 68.1 ± 10.5 |
| Male, n (%) | 54 (65.1) |
| Ever smokers, n (%) | 50 (60.2) |
| Height, cm | 162.3 ± 8.5 |
| Weight, kg | 64.7 ± 10.9 |
| BMI, kg/m^2^ | 24.5 ± 3.4 |
| Home O_2_, n (%) | 5 (6.0) |
| Interstitial lung disease |  |
| IPF, n (%) | 57 (68.7) |
| CTD, n (%) | 12 (14.5) |
| COP, n (%) | 8 (9.6) |
| NSIP, n (%) | 5 (6.0) |
| HP, n (%) | 1 (1.2) |
| Radiologic pattern |  |
| UIP, n (%) | 56 (67.4) |
| NSIP, n (%) | 10 (12.1) |
| OP, n (%) | 10 (12.1) |
| Indeterminate, n (%) | 6 (7.2) |
| Pulmonary function |  |
| FVC, % predicted | 73.9 ± 15.6 |
| DLco, % predicted | 59.1 ± 17.0 |
| TLC, % predicted | 72.5 ± 12.4 |
| FEV1/FVC, % | 77.0 ± 8.5 |
| Six-minute walk test |  |
| Distance, m | 402.2 ± 99.0 |
| Baseline SpO_2,_ % | 95.9 ± 2.5 |
| Lowest SpO_2,_ % | 90.6 ± 5.9 |
| BAL |  |
| Total WBC count, cell/μL | 2,135.7 ± 3,402.5 |
| Neutrophil_,_ % | 28.6 ± 26.3 |
| Lymphocyte_,_ % | 18.6 ± 21.8 |
| PaO_2,_ mmHg | 88.0 ± 28.2 |
| BNP, pg/mL | 421.1 ± 734.5 |
| Baseline IL-6, pg/mL | 14.9 ± 33.8 |
| CRP, mg/dL | 2.5 ± 5.5 |
| WBC, cell/μL | 8,094.8 ± 3,041.7 |
| LDH, IU/L | 264.8 ± 86.3 |

Values are presented as mean ± standard deviation or number (%)

**Table 2. Comparison of Characteristics Between ILD patients With and Without AE**

| Variable | AE (+) | No AE(-) | *P*-value |
| --- | --- | --- | --- |
|  | (n = 15) | (n = 68) |  |
| Number of patients, n (%) | 15 (18.1) | 68 (81.9) |  |
| Age, years | 70.3 ± 5.8 | 67.6 ± 11.2 | .477 |
| Male, n (%) | 10 (66.7) | 44 (64.7) | .885 |
| Ever smokers, n (%) | 7 (46.7) | 43 (64.2) | .209 |
| BMI, kg/m^2^ | 23.9 ± 4.1 | 24.6 ± 3.3 | .368 |
| Interstitial lung disease |  |  |  |
| IPF, n (%) | 12 (80.0) | 45 (66.2) | .296 |
| CTD, n (%) | 3 (20.0) | 9 (13.2) | .500 |
| Pulmonary function |  |  |  |
| FVC, % predicted | 69.5 ± 13.1 | 74.9 ± 15.9 | .219 |
| DLco, % predicted | 52.5 ± 16.8 | 60.5 ± 16.8 | .179 |
| TLC, % predicted | 76.0 ± 13.4 | 71.6 ± 12.4 | .536 |
| Six-minute walk test |  |  |  |
| Distance, m | 355.9 ± 101.9 | 410.8 ± 96.8 | .084 |
| Baseline SpO_2,_ % | 94.3 ± 2.3 | 96.2 ±2.4 | .009 |
| Lowest SpO_2,_ % | 88.8 ± 5.5 | 90.9 ± 5.9 | .153 |
| Baseline IL-6, pg/mL | 35.5 ± 68.3 | 10.5 ± 17.5 | .057 |
| Peak IL-6*, pg/mL | 133.5 ± 205.9 | 21.1 ± 35.0 | <.001 |
| CRP, mg/dL | 7.9 ± 9.9 | 1.2 ± 2.5 | <.001 |
| WBC, cell/μL | 9,614.0 ± 4,169.3 | 7,759.7 ± 2,657.2 | .024 |
| LDH, IU/L | 317.0 ± 147.7 | 255.1 ± 67.0 | .226 |

Q2. Figure 2: survival analysis, please correct mislabeling (IL6 level).

R1. Thank you for your comment. Sorry for our mistake. This is a typing error, and was corrected as below.

Q3. As a predictive factor for AE in the CTD-group, n=3 is too small for biomarker study. I agree to the reviewer’s comments to remove CTD group from study.

R1. Thank you for pointing this out. We agree to your comment. The paragraph “Predictive factor for AE in the CTD-group” removed from the manuscript.

**Journal Requirements**

Q1. Please ensure that your manuscript meets PLOS ONE's style requirements, including those for file naming.

R1. Thank you for pointing this out. Based on this comment, we ensured that our manuscript meets PLOS ONE's style requirements.

Q2. PLOS requires an ORCID iD for the corresponding author in Editorial Manager on papers submitted after December 6th, 2016. Please ensure that you have an ORCID iD and that it is validated in Editorial Manager. To do this, go to ‘Update my Information’ (in the upper left-hand corner of the main menu), and click on the Fetch/Validate link next to the ORCID field. This will take you to the ORCID site and allow you to create a new iD or authenticate a pre-existing iD in Editorial Manager.

R2. Thank you for pointing this out. We updated an ORCID iD for the corresponding author in Editorial Manager.

Q3. Thank you for stating in the text of your manuscript "the requirement for written informed consent was waived due to the retrospective nature of this study". Please also add this information to your ethics statement in the online submission form."

R3. Thank you for pointing this out. We add the statement "the requirement for written informed consent was waived due to the retrospective nature of this study" in the online submission form.

Q4. Thank you for providing the date(s) when patient medical information was initially recorded. Please also include the date(s) on which your research team accessed the databases/records to obtain the retrospective data used in your study.

R4. Thank you for pointing this out. We added the date in medical records in Clinical information of MATERIALS AND METHODS as below.

[MATERIALS AND METHODS, Clinical information]

Clinical data were retrospectively obtained from the medical records at January 3, 2020.

Q5. Thank you for stating the following in the Acknowledgments Section of your manuscript:

This work was supported by the 2019 Inje University Research Grant (20190041).

We note that you have provided funding information that is not currently declared in your Funding Statement. However, funding information should not appear in the Acknowledgments section or other areas of your manuscript. We will only publish funding information present in the Funding Statement section of the online submission form.

Please remove any funding-related text from the manuscript and let us know how you would like to update your Funding Statement. Currently, your Funding Statement reads as follows:

This study was supported by Inje university research grant.  The grant has nothing to do with the result of this research and is only to encourage the research.

Please include your amended statements within your cover letter; we will change the online submission form on your behalf.

R5. Thank you for pointing this out. We deleted this sentence in the Acknowledgments and also added the Funding Statement section of the online submission form.

**Reviewer 1**

Q1. Figure 2. Is the labels for “Peak IL-6 level>25.20” and “Peak IL-6 level<25.20” flipped?

R1. Thank you for your comment. Sorry for our mistake. This is a typing error, and was corrected as below.

**Reviewer 2**

Q1. Limitation: an important limitation of the paper is the low number of patients, with probable insufficient power (the power of the study sample needs to be determined) and the heterogeneity of included diseases Moreover, the OR and HR of IL6 to predict the occurrence of AE were low, close to 1, the question is their relevance in clinical pertinence

R1. Thank you for pointing this out. I agree with your opinion. In this study, the value of OR of IL-6 to predict to acute exacerbation and mortality was 1.014 (95% CI: 1.001 – 1.027) and 1.007 (95%CI: 1.001 – 1.014), respectively. However, the *P* – value was statistically significant. The diagnosis of acute exacerbation is still difficult and very important for good prognosis. Although revised diagnostic criteria of AE IPF, early and accurate diagnosis of AE ILD is still big challenging. In our county, other biomarker including KL-6 and SP-D had not been avilable. Therefore, we evaluated the role of IL-6 to predict AE. As you know, there cannot be a perfect biomarker. We expect that IL-6 might be helpful to predict to AE in addition to other method. Also IL-6 can be more useful as an acute reactant than other biomarker.

Q2. Please precise the number of screened patients, those who fulfilled inclusion and exclusion criteria leading to the final number of recruited patients

R2. Thank you for pointing this out. According to reviewer’s comment, we added flow-chart for enrollment (Fig 1) in Study population of RESUTLS as below

[Study population in RESULTS]

From December 2016 to September 2019, 405 patients with ILD at Haeundae Paik hospital (Busan, Republic of Korea) were screening for this study. Among them, patients who did not perform IL-6 test or follow up at IL-6 test at least 6 months were excluded (Fig 1).

Q3. The ILD pattern on HRCT scan should me precised: UIP, NSIP…

R3. Thank you for pointing this out. We added Radiologic pattern on HRCT to the table 1 as below.

**Table 1. Baseline Clinical Characteristics of the Patients**

| **Characteristics** | **All Patients (n = 83)** |
| --- | --- |
| Age, years | 68.1 ± 10.5 |
| Male, n (%) | 54 (65.1) |
| Ever smokers, n (%) | 50 (60.2) |
| Height, cm | 162.3 ± 8.5 |
| Weight, kg | 64.7 ± 10.9 |
| BMI, kg/m^2^ | 24.5 ± 3.4 |
| Home O_2_, n (%) | 5 (6.0) |
| Interstitial lung disease |  |
| IPF, n (%) | 57 (68.7) |
| CTD, n (%) | 12 (14.5) |
| COP, n (%) | 8 (9.6) |
| NSIP, n (%) | 5 (6.0) |
| HP, n (%) | 1 (1.2) |
| Radiologic pattern |  |
| UIP, n (%) | 56 (67.4) |
| NSIP, n (%) | 10 (12.1) |
| OP, n (%) | 10 (12.1) |
| Indeterminate, n (%) | 6 (7.2) |
| Pulmonary function |  |
| FVC, % predicted | 73.9 ± 15.6 |
| DLco, % predicted | 59.1 ± 17.0 |
| TLC, % predicted | 72.5 ± 12.4 |
| FEV1/FVC, % | 77.0 ± 8.5 |
| Six-minute walk test |  |
| Distance, m | 402.2 ± 99.0 |
| Baseline SpO_2,_ % | 95.9 ± 2.5 |
| Lowest SpO_2,_ % | 90.6 ± 5.9 |
| BAL |  |
| Total WBC count, cell/μL | 2,135.7 ± 3,402.5 |
| Neutrophil_,_ % | 28.6 ± 26.3 |
| Lymphocyte_,_ % | 18.6 ± 21.8 |
| PaO_2,_ mmHg | 88.0 ± 28.2 |
| BNP, pg/mL | 421.1 ± 734.5 |
| Baseline IL-6, pg/mL | 14.9 ± 33.8 |
| CRP, mg/dL | 2.5 ± 5.5 |
| WBC, cell/μL | 8,094.8 ± 3,041.7 |
| LDH, IU/L | 264.8 ± 86.3 |

Values are presented as mean ± standard deviation or number (%)

BMI, body mass index; IPF, idiopathic pulmonary fibrosis; CTD, connective tissue disease-associated interstitial lung disease; COP, cryptogenic organizing pneumonia; NSIP, non-specific interstitial pneumonia; HP, hypersensitivity pneumonitis; UIP, usual interstitial pneumonia; OP, organizing pneumonia; FVC, forced vital capacity; DLco, diffusing capacity of the lungs for carbon monoxide; TLC, total lung capacity; SpO2, saturation of peripheral oxygen; BAL, bronchoalveolar lavage; WBC, white blood cell; PaO2, partial pressure of oxygen; BNP, brain natriuretic peptide; IL-6, interleukin-6; CRP, C-reactive protein; LDH, lactate dehydrogenase

Q4. Please provide the detailed causes of AE. Please confirm that infections have been formally ruled out. It is important since infections may increase CRP and thus, IL-6 levels.

R4. Thank you for pointing this out. As a result of re-analysis, 11 patients and 4 patients were reclassified as idiopathic and triggered AE, respectively. Triggered AE was defined as proven pathogen or increased level of procalcitonin. We added detail data in Predictive factors in RESULTS as below.

[RESULTS, Predictive factors]

Among the 15 (18.1%) patients with AE (idiopathic AE: 11 patients and triggered AE: 4 patients), IPF patients were most common (80%) while the other patients were classified with CTD (20%).

Q5. Logistic regression analysis: please explain how were entered the different covariates in the model (stepwise, backward…). In this model, IL-6 was entered as a continuous variable; it would be interesting to consider IL-6 as a dichotomous variable (Lower/higher)

R5. Thank your comment. We modified the sentence in Statistical analysis as below.

[MATERIALS AND METHODS, Statistical analysis]

Multivariate analyses, using Cox regression with backward and stepwise elimination, were performed in order to identify prognostic factors which are independently related to mortality.

Q6. Multivariate analyses: gender, disease duration, and HRCT scan pattern should be included in the models

R6. Thank you for comment. According to comment, we added the variables including gender, disease duration and radiologic pattern as below. However, NSIP, OP and indeterminate pattern were excluded from multivariate analysis because they caused statistical error.

**Table 3. Predictive Factors for AE in ILD Patients Assessed using the Logistic Regression Model**

| Variable | Univariate Analysis | | Multivariate Analysis | |
| --- | --- | --- | --- | --- |
|  | OR (95% CI) | *P*-value | OR (95% CI) | *P*-value |
| Age | 1.028 (0.969 - 1.091) | .362 |  |  |
| Male | 1.091 (0.034-3.561) | .885 |  |  |
| Ever smokers | 0.488 (0.158 - 1.513) | .214 |  |  |
| BMI | 0.942 (0.791 - 1.122) | .501 |  |  |
| Disease duration | 1.031 (1.005-1.057) | .017 |  |  |
| Interstitial lung disease |  |  |  |  |
| IPF | 0.489 (0.125 - 1.908) | .303 |  |  |
| CTD | 0.610 (0.144 – 2.592) | .503 |  |  |
| Radiologic pattern |  |  |  |  |
| UIP | 0.115 (0.014 – 0.930) | .430 |  |  |
| Pulmonary function |  |  |  |  |
| FVC | 0.995 (0.940 - 1.014) | .218 |  |  |
| DLco | 0.969 (0.934 - 1.006) | .104 |  |  |
| TLC | 1.032 (0.939 - 1.134) | .516 |  |  |
| Six-minute walk test |  |  |  |  |
| Distance | 0.995 (0.989 - 1.001) | .084 |  |  |
| Baseline SpO_2_ | 0.738 (0.573 - 0.952) | .019 | 0.750 (0.568 - 0.991) | .043 |
| Lowest SpO_2_ | 0.948 (0.862 - 1.042) | .265 |  |  |
| PaO_2_ | 0.994 (0.969 - 1.019) | .628 |  |  |
| RVSP | 1.095 (1.001 - 1.199) | .048 |  |  |
| Baseline IL-6 | 1.018 (0.999 - 1.037) | .062 |  |  |
| ^*^Peak IL-6 | 1.017 (1.006 - 1.028) | .002 | 1.014 (1.001 - 1.027) | .036 |
| CRP | 1.223 (1.054 - 1.442) | .009 |  |  |
| WBC | 1.000 (1.000 - 1.000) | .045 |  |  |
| LDH | 1.007 (1.000 - 1.013) | .049 |  |  |

**Table 4. Prognostic Factors for Mortality in Patients with ILD Assessed Using Cox Proportional Hazards Model**

| Variable | Univariate Analysis | | Multivariate Analysis | |
| --- | --- | --- | --- | --- |
|  | HR (95% CI) | *P*-value | HR (95% CI) | *P*-value |
| Age | 1.211 (1.002 - 1.465) | .048 |  |  |
| Male | 0.206 (0.021 – 1.993) | .172 |  |  |
| Ever smokers | 0.016 (0.000 - 51.983) | .317 |  |  |
| BMI | 0.746 (0.504 - 1.104) | .143 |  |  |
| Disease duration | 0.810 (0.581 – 1.130) | .215 |  |  |
| Interstitial lung disease |  |  |  |  |
| IPF | 0.667 (0.068 - 6.505) | .728 |  |  |
| CTD | 0.857 (0.089 - 8.294) | .894 |  |  |
| Radiologic pattern |  |  |  |  |
| UIP | 0.031 (0.001 – 598.0) | .491 |  |  |
| Pulmonary function |  |  |  |  |
| FVC | 1.031 (0.936 - 1.135) | .538 |  |  |
| DLco | 1.011 (0.946 - 1.081) | .742 |  |  |
| TLC | 1.177 (0.903 - 1.535) | .229 |  |  |
| Six-minute walk test |  |  |  |  |
| Distance | 0.988 (0.976 - 0.999) | .037 |  |  |
| Baseline SpO_2_ | 0.802 (0.502 - 1.280) | .355 |  |  |
| Lowest SpO_2_ | 0.992 (0.809 - 1.217) | .938 |  |  |
| PaO_2_ | 0.998 (0.929 - 1.072) | .955 |  |  |
| Baseline IL-6 | 0.992 (0.953 - 1.033) | .694 |  |  |
| ^*^Peak IL-6 | 1.007 (1.001 - 1.014) | .018 | 1.007 (1.001 - 1.014) | .018 |
| CRP | 0.914 (0.640 - 1.305) | .620 |  |  |
| LDH | 0.999 (0.985 - 1.013) | .882 |  |  |

Q7. The pertinence to include in the models both CRP and IL-6 is questionable given the strong relationship between these 2 variables

R7. Thank you for your comment. CRP is produced by hepatocyte in the liver in response to stimulation of pro-inflammatory cytokines. Therefore, CRP has been used as biomarker for inflammation. In several study, elevated level of CRP was reported as useful biomarker to predict AE ILD. In this study, baseline level of CRP was significantly different between AE group and non-AE group. However, high level of CRP was not useful to predict AE and mortality, although IL-6 was useful to predict both. In this study, the role of CRP and IL-6 was different to predict AE and mortality. In the field of ILD, we thought the role of CRP and IL-6 are different and independent.

Q8. Kaplan-Meier survival curve: a table with the number of patients should be added below the figure

R8. Thank you for your comment. We added a table with the number of patients in Figure 3 as below.

Q9. Given the very low number of patients with CTD-ILD, and the low frequency of AE, the reviewer suggests the deletion of the sub analysis of the CTD-group

R9. Thank you for your comment. The paragraph “Predictive factor for AE in the CTD-group” was removed from the manuscript.

Q10. How were Handled missing data?

R10. Thank you for comment. There were some missing data. Therefore, the missing data was null out and the remaining data was analyzed. Variables with many missing data such as RVSP, were excluded from the statistical analysis because they caused statistical error.
